# Hydroxychloroquine-Induced Hepatotoxicity in Systemic Lupus Erythematous: A Case Report and Literature Review

**DOI:** 10.7759/cureus.81664

**Published:** 2025-04-03

**Authors:** Dana Chen, Catherine Pham, Mandeep Kaur, Kevin Yip

**Affiliations:** 1 School of Osteopathic Medicine, A.T. Still University, Mesa, USA; 2 School of Medicine, St. George’s University, St. George’s, GRD; 3 Internal Medicine, Wyckoff Heights Medical Center, New York City, USA; 4 Rheumatology, Wyckoff Heights Medical Center, New York City, USA

**Keywords:** drug-induced liver injury, hepatitis, hepatotoxicity, hydroxychloroquine, systemic lupus erythematous

## Abstract

Hydroxychloroquine (HCQ) is a medication that is commonly used as an antimalarial or disease-modifying anti-rheumatic drug (DMARD). Adverse side effects typically include retinal damage, cardiomyopathy, and neuromyopathy. However, there has been relatively little documentation on the effects of HCQ toxicity on the liver. We describe a case of HCQ-induced hepatic failure in a 31-year-old female patient on HCQ for systemic lupus erythematous who presented with three days of fever, diarrhea, and non-bilious, non-bloody vomiting. Labs showed massively elevated liver function tests (LFTs) and negative viral serology. The abdominal ultrasound and magnetic resonance cholangiopancreatography were unremarkable. A liver biopsy showed portal tracts with mild to moderate expansion by mixed inflammatory infiltrates, including scattered eosinophils and rare plasma cells with occasional mild interface activity focally close to bridging inflammation. These findings are consistent with drug-induced liver injury (DILI). HCQ was subsequently held, and the patient was admitted to the ICU for conservative management. Repeat LFTs showed down-trending over the course of the patient’s four days of admission after discontinuation of HCQ and returned to baseline within a two-month timeframe.

## Introduction

Hydroxychloroquine (HCQ), a medication under the 4-aminoquinoline class of drugs, was first introduced as a treatment for malaria but is now also widely used in the management of autoimmune diseases [1]. HCQ has a relatively favorable benefit/risk ratio and is one of the most cost-effective drugs in rheumatology due to its extensive therapeutic effects in multiple systemic diseases [2]. Despite its benefits, HCQ has various commonly recognized rare side effects, such as cardiomyopathy, neuromyopathy, hematological abnormalities, and dermatological symptoms [3]. Relative to the other adverse side effects mentioned, HCQ-associated hepatotoxicity is rare, with only a few reports recognizing side effects such as fulminant hepatic failure, although the pathophysiological mechanisms are not as well understood [4]. For the literature review portion of this report, we used the terms “hydroxychloroquine” and “hepatotoxicity” as our inclusion criteria on PubMed Central and the Consensus academic search engine. In this case report, we describe HCQ-induced hepatotoxicity, a rare yet potentially serious adverse effect of HCQ that requires further investigation and should be considered as a differential diagnosis in patients with a history of connective tissue diseases and acute liver issues.

## Case presentation

This patient is a 31-year-old female individual with a past medical history significant for gastroesophageal reflux, uveitis, and systemic lupus erythematosus (SLE), which was diagnosed two months prior to presentation with positive anti-nuclear antibodies (ANA), anti-doube stranded DNA (anti-dsDNA), and anti-Smith antibodies after having initial findings of uveitis, joint pain, and hair loss. One month later, she was started on hydroxychloroquine 200 mg twice per day.

**Figure 1:**
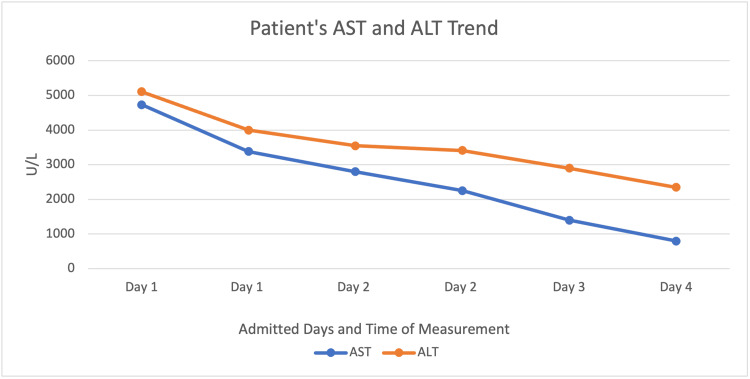
Patient’s serum levels of aspartate aminotransferase (AST) and alanine aminotransferase (ALT) over the course of her four days admitted in the hospital.

A month after hydroxychloroquine initiation, she developed abdominal pain and vomiting. Subsequently, she took one dose of acetaminophen, dextromethorphan, and phenylephrine in the morning and one dose in the evening at the recommended dosage. The following day, at urgent care, she was diagnosed with viral gastritis and received one gram of acetaminophen. Three days later, she presented to the emergency room with acute liver failure, with peak aspartate aminotransferase (AST) 4731 U/L and alanine aminotransferase (ALT) 5109 U/L (Figure 1).

No obvious acute inciting event has been found for the elevated AST and ALT levels. She denied any chest pain, dizziness, palpitations, cough, joint pains, or skin rashes. The patient denied any side effects since starting hydroxychloroquine. Her medications included hydroxychloroquine 200 mg twice per day, pantoprazole 40 mg one tablet daily, meloxicam 15 mg one tablet as required, brimonidine/timolol 0.2%-0.5% ophthalmic solution, dorzolamide/timolol 2%-0.5% ophthalmic solution, dorzolamide ophthalmic, and prednisolone ophthalmic as prescribed. She denied any other over-the-counter medications, illicit drug use, or other unknown intoxicants. She endorsed previous marijuana use but has not used for the past six months. The patient also endorsed minimal social alcohol use, with the last alcohol intake roughly two weeks prior to hospitalization.

On physical examination, the patient was alert and oriented. She was afebrile, blood pressure was 120/78, heart rate was 70 beats per minute, respiratory rate was 19, and oxygen saturation was 99% on room air. Her respiratory and cardiac exams were normal. There was no lymphadenopathy or pitting edema noted. She had epigastric abdominal pain, diarrhea, nausea, and vomiting, but no ascites. There was no evidence of active synovitis in any of her joints and no skin rashes or jaundice to note. On global assessment, her lupus activity appeared to be quiescent, with unremarkable lupus activity markers such as normal complement levels.

Our patient was admitted to the intensive care unit for close monitoring. The patient was briefly started on ceftriaxone, fluconazole, metronidazole, dextrose 5%, lactated Ringer’s, and one dose of N-acetylcysteine. She also received an abdominal ultrasound, CT abdomen, magnetic resonance cholangiopancreatography (MRCP), liver biopsy, hepatitis serology, and HIV test. She was checked for acetaminophen levels, alcohol levels, and daily liver function tests (LFTs). Her hydroxychloroquine was immediately held.

**Figure 2:**
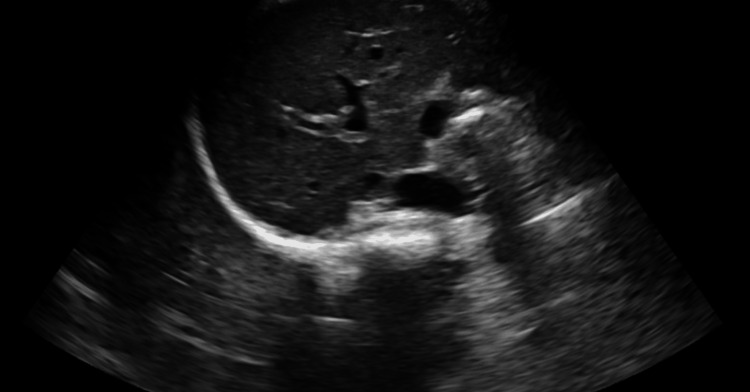
Abdominal ultrasound demonstrating mild gallbladder wall thickening; nonspecific, and no gallstones.

The patient’s initial lab work was remarkable for AST 4731 U/L, ALT 5109 U/L, alkaline phosphatase 391 U/L, direct bilirubin 1.44 mg/dL, total bilirubin 2.0 mg/dL, lactate dehydrogenase 909 U/L, ferritin 14800 ug/L, C3 93 mg/dL, C4 37 mg/dL, creatine phosphokinase (CPK) 70 U/L, prothrombin time 16.8 seconds, partial thromboplastin time 29.7 seconds, hemoglobin 12.6 g/dL, 127 x 109 platelets/L, 2.62 x 109 white blood cells/L, and normal renal function. Urinalysis showed urine protein of 100 mg and a lack of red blood cells. Testing for hepatitis A, hepatitis B, hepatitis C, HIV, and cytomegalovirus (CMV) were all negative, as well as acetaminophen, lead, and ethanol toxicity. An abdominal ultrasound demonstrated mild gallbladder wall thickening; nonspecific, and no gallstones (Figure 2).

**Figure 3:**
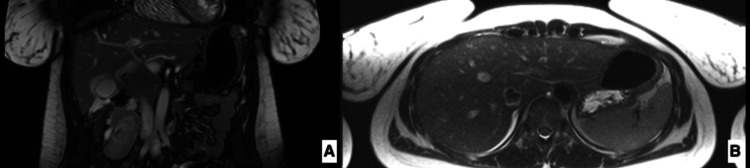
Magnetic resonance cholangiopancreatography (MRCP) demonstrating non-obstructive hepatobiliary ductal system, normal common bile duct, no choledocholithiasis, and possible inflammatory etiology which may correlate with intrinsic hepatic disease. (A) Coronal view, (B) axial view.

Abdominal CT was suspicious for gallbladder wall inflammation and normal portal vein. MRCP showed a non-obstructive hepatobiliary ductal system, a normal common bile duct, no choledocholithiasis, and possible inflammatory etiology, which may correlate with intrinsic hepatic disease (Figure 3).

**Figure 4:**
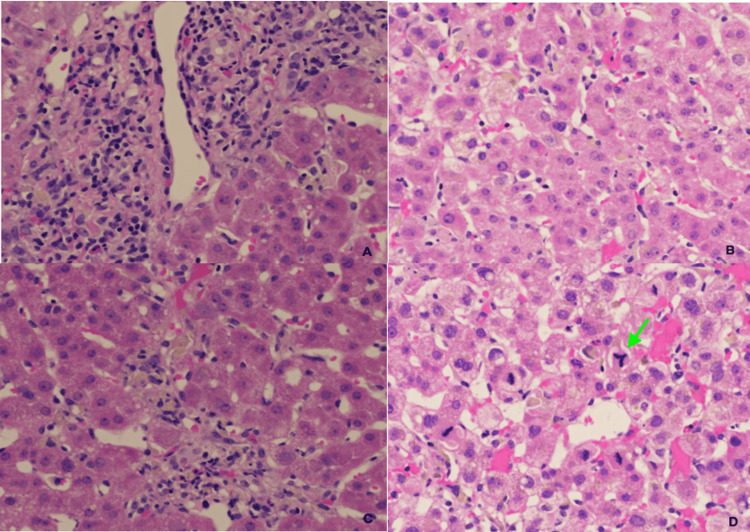
Liver biopsy demonstrating moderate hepatitis consistent with drug-induced liver injury pattern. (A) Mild to moderate expansion of portal tracts by mixed inflammatory infiltrates including scattered eosinophils and rare plasma cells; (B) Kupffer cell hyperplasia and abundant ceroid/pigment containing macrophage; (C) Perivenular confluent necrosis; (D) Numerous mitotic figures and note the tripolar mitotic figure on the right (green arrow).

Liver biopsy demonstrated moderate hepatitis consistent with a drug-induced liver injury (DILI) pattern (Figure 4). Liver parenchyma showed preserved hepatic architecture and diffuse lobular activity as characterized by mild lymphocytic inflammation, Kupffer hyperplasia, abundant ceroid pigment-containing macrophage, foci of necroinflammation, occasional perivenular confluent necrosis, and apoptotic hepatocytes, along with mitotic activity and regeneration of hepatocytes. Portal tracts showed mild to moderate expansion by mixed inflammatory infiltrates, including scattered eosinophils and rare plasma cells with occasional mild interface activity focally close to bridging inflammation. Bile ducts are preserved or obscured by portal inflammation. There was no steatosis and no overt cholestasis. Trichrome stain showed no fibrosis. An iron stain was negative. Periodic acid-Schiff-diastase stain highlighted lobular inflammation and abundant ceroid-/pigment-containing macrophages. Reticulin stain highlighted patchy hepatic plates of 2-3 cell thickness. CK7 immunochemistry stain showed bile ducts, mild ductular reaction, and rare metaplastic hepatocytes. The findings are those of acute moderate hepatitis compatible with DILI.

The patient was discharged to outpatient follow-up after being admitted for four days in the hospital for conservative management and observation. The patient's LFTs were repeated; AST downtrended from 4731 U/L to 796 U/L, and ALT downtrended from 5109 U/L to 2346 U/L by the end of her hospital stay. On follow-up in the outpatient setting, the LFTs had normalized within a two-month time period.

## Discussion

Hydroxychloroquine was first synthesized in 1950 and later approved by the United States Food and Drug Administration for medical use in 1955 [1]. Initially introduced as a treatment for malaria, HCQ is now also widely used in the management of connective tissue diseases such as systemic lupus erythematous, with various studies supporting its efficacy [4,5]. The therapeutic dose for HCQ in treating SLE is 5 mg/kg, and extended use at high doses can result in HCQ toxicity [6]. Despite its established use in medicine, HCQ still has various rare yet detrimental side effects that are not well understood. In this case report, we discuss our patient with SLE who developed adverse gastrointestinal symptoms and drastically elevated LFTs within a few weeks of HCQ initiation.

Hepatotoxicity due to HCQ is very rare but has been documented. In reviewing previously published reports, we found eight other cases of HCQ-induced liver toxicity. In one of the earliest case reports, Makin et al. described two cases of patients who both developed fulminant hepatic failure within two weeks of HCQ initiation [7]. In another case, Sunkara et al. described hydroxychloroquine-induced liver injury in a patient with subacute cutaneous lupus erythematous and porphyria cutanea tarda overlap syndrome [8]. Existing case reports demonstrated a predisposition for female patients ranging from 16 to 62 years old, with a mean age of onset of 31.6 years old [7-13] (Table 1). Among the eight patients, the following conditions were the indications for HCQ use: SLE, juvenile-onset Still’s disease, adult-onset Still’s disease, mixed connective tissue disease, and coronavirus disease 2019. Most of the patients had no history of any pre-existing hepatic disease. One patient had a history of rheumatoid arthritis, and another patient had a diagnosis of subacute cutaneous lupus erythematous and porphyria cutanea tarda overlap syndrome. Five out of the eight patients were also being treated with either prednisolone or prednisone at the time of presentation. The mean cumulative dose of HCQ used to treat the patients was 342 mg daily, and the mean duration of treatment prior to the onset of liver injury symptoms was 53.8 days. The mean AST was 1160 U/L, and the mean ALT was 1880 U/L. Most of the patients had a similar presentation of fever, nausea, vomiting, abdominal pain, jaundice, and generalized fatigue. One patient expired prior to liver transplantation, another patient expired post-liver transplantation, and the remaining six patients recovered back to baseline after discontinuing HCQ. Three of the patients who recovered were also treated with a glucocorticoid in addition to stopping HCQ. Fitting into the demographic profile of the above-mentioned cases of HCQ-induced hepatotoxicity, our female patient developed acute liver injury at age 31 after one month of exposure.

**Table 1:** Demographic features and clinical characteristics of all nine currently known cases, including our patient, of hydroxychloroquine-induced hepatotoxicity. HCQ, hydroxychloroquine; SLE, systemic lupus erythematosus; AST, aspartate aminotransferase; ALT, alanine aminotransferase; -, not mentioned in case report.

	Our case	Makin et al. (Case 1) [7]	Makin et al. (Case 2) [7]	Giner-Galvañ et al. [9]	Abdel Galil et al. [10]	Sunkara et al. [8]	Cheema et al. [13]	Falcão et al. [11]	Gisi et al. [12]
Age (years)	31	27	16	26	28	29	62	29	36
Sex	Female	Female	Female	Female	Female	Female	Female	Female	Female
Disease or HCQ indication	SLE	SLE	Juvenile Still’s disease	Mixed connective tissue disease	SLE	Subacute cutaneous lupus erythematous	-	COVID-19	Adult Still’s disease
Other pertinent medical history	No pre-existing liver disease	No pre-existing liver disease	No pre-existing liver disease	No pre-existing liver disease	No pre-existing liver disease	Porphyria cutanea tarda	Rheumatoid arthritis	-	-
Other medications	Acetaminophen 1 mg, dextromethorphan, phenylephrine, doxylamine, pantoprazole 40 mg once daily, meloxicam 15 mg once daily	Prednisolone 30 mg, ibuprofen 400 mg twice daily (previous year)	Indomethacin 50 mg three times daily	Prednisone 40 mg once daily	Prednisolone 25 mg once daily (tapered down from 40 mg)	None	Prednisone	Azithromycin, piperacillin-tazobactam	Prednisolone 10 mg once daily
HCQ dosage	200 mg twice daily	200 mg twice daily	200 mg twice daily	200 mg once daily	400 mg once daily	400 mg once daily	-	400 mg twice daily	200 mg once daily
Duration between HCQ initiation and onset of adverse side effects	1 month	2 weeks	2 weeks	8-10 hours	1 year	3 days	12 days	1 day	3 weeks
Adverse side effects and symptoms	Fever, abdominal pain, vomiting, diarrhea	Nausea, vomiting, increasing jaundice, encephalopathy, cerebral edema, sepsis, hypotension	Increasing jaundice, general malaise, arthritis flare-up, encephalopathy, cerebral edema	Fever, nausea, vomiting	Abdominal pain, nausea, vomiting, diarrhea	Nausea, fatigue, chills, malaise, dark urine	Arthralgias, generalized weakness, fever	-	Jaundice, abdominal pain
AST (U/L)	4731	-	544	399	745	4472	798	469	693
ALT (U/L)	5109	2575	-	285	987	6380	1254	357	1325
Total bilirubin (mg/dL)	2	17.661	24.386	-	-	-	Normal	-	22.3
Liver biopsy	Mild lymphocytic inflammation, Kupffer hyperplasia, abundant ceroid pigment-containing macrophage, foci of necroinflammation, occasional perivenular confluent necrosis, and apoptotic hepatocytes, along with mitotic activity and regeneration of hepatocytes. Portal tracts show mild to moderate expansion by mixed inflammatory infiltrates, including scattered eosinophils and rare plasma cells with occasional mild interface activity focally close to bridging inflammation	Sub-massive necrosis of the parenchyma with near complete hepatocyte dropout and few duct-like structures in periportal areas	Massive necrosis of the parenchyma with confluent multi-acinar cell dropout. Moderately heavy inflammatory infiltrate throughout with pigment-laden macrophages, eosinophils, and increased plasma cell count	-	Patient refused	Early bridging fibrosis and nodularity, consistent with post-necrotic cirrhosis	-	-	Hydropic degeneration, edema, rosette-like appearance, lymphocyte, polymorphous leukocyte, spotty necrosis, and bridging confluent necrosis areas, consistent with acute toxic hepatitis
Treatment for HCQ-induced hepatotoxicity and symptoms	HCQ discontinued. IV ceftriaxone, IV metronidazole, N-acetylcysteine, and IV fluids	None (patient was awaiting urgent liver transplantation)	Urgent liver transplantation	HCQ discontinued. IV methylprednisolone 60 mg once daily and IV ceftriaxone 1 g once daily	HCQ discontinued and prednisolone tapered down. IV fluids with paracetamol 500 mL for 2 hours, dompridone 10 mg three times daily for 2 weeks, and mycophenolate mofetil 2 g daily	HCQ discontinued. Therapeutic phlebotomy	HCQ discontinued	HCQ discontinued	HCQ discontinued. Methylprednisolone 40 mg once daily
Outcome	Recovered back to baseline	Expired	Expired	Recovered back to baseline	Recovered back to baseline	Recovered back to baseline	Recovered back to baseline	Recovered back to baseline	Recovered back to baseline

HCQ interferes with enzymatic activity within the endolysosomal compartment, impairs antigen processing and presentation, and ultimately blunts the immune response [1, 14]. Additionally, HCQ plays a role in the inhibition of TLR-7, TLR-9, cyclic GMP-AMP synthase activity, IL-1β, IL-6, and TNF-α [4]. All of these actions help promote the anti-inflammatory effects of HCQ, which proves to be efficacious in treating diseases like SLE. However, HCQ’s diverse mechanisms of action can also explain its undesirable systemic toxicity. It can alter normal physiological functions such as hemostasis, tumor control, lipid metabolism, and glucose metabolism [15].

The pathophysiological mechanism underlying HCQ hepatotoxicity is poorly understood. However, it is known that HCQ is heavily metabolized by cytochrome P450 in the liver, which may indirectly play a part in the liver damage process [1]. Chloroquine, which also falls under the 4-aminoquinoline class of drugs, may share a similar pathophysiologic mechanism of toxicity as HCQ due to their similar molecular structure [1]. In an in vivo study, Niknahad et al found that rats treated with both bacterial lipopolysaccharide, which induces a modest inflammatory response, and chloroquine later developed signs of liver injury; this suggests that the quinolone class of drugs can potentially induce liver injury in those with concurrent inflammatory conditions such as malaria [16]. Thus, we may need to also consider the possible synergistic effect of hydroxychloroquine and inflammatory connective tissue diseases in the development of hepatotoxicity in these patients. To the author’s best knowledge, there have been no randomized controlled studies examining hydroxychloroquine-induced acute liver injury in humans.

It is important to highlight that hepatic injury in patients with a history of SLE can be attributed to either HCQ toxicity or SLE-related liver disease. Patients may present with elevated liver enzymes, which is a relatively common finding in those with SLE. Some hepatic diseases that can be found in patients with SLE include chronic hepatitis, steatosis, primary biliary cholangitis, hepatic arteritis, autoimmune hepatitis, and nodular regenerative hyperplasia [17]. Lupus hepatitis, which is strongly associated with anti-ribosomal P antibodies, is a rare yet distinct hepatic manifestation of SLE [18] (Table 2). However, lupus hepatitis is improbable in our patient, given the otherwise quiescent state of her SLE. Additionally, end-stage liver disease is relatively rare in SLE patients unless they have pre-existing hepatic disorders such as autoimmune hepatitis, non-alcoholic fatty liver disease, or viral hepatitis [19].

**Table 2:** Differential diagnoses for acute liver injury in patients with systemic lupus erythematous (SLE). EBV, Epstein-Barr virus; CMV, cytomegalovirus.

Differential diagnoses for acute liver injury in patients with SLE
Autoimmune hepatitis
EBV-associated hepatitis
Lupus hepatitis
Hepatitis A, B, and C
Acute viral hepatitis
Wilson’s disease
CMV-associated hepatitis
Cholestatic liver disease
Ischemic hepatic failure
Drug-induced liver injury

## Conclusions

In SLE patients presenting with acute liver failure, a broad differential must be considered, including autoimmune hepatitis, Epstein-Barr virus (EBV)-associated hepatitis, DILI, lupus hepatitis, hepatitis A, hepatitis B, hepatitis C, acute viral hepatitis, Wilson’s disease, CMV-associated hepatitis, cholestatic liver disease, and ischemic hepatic failure. Other causes of a non-liver origin elevation of AST and ALT include rhabdomyolysis and myositis, which were effectively ruled out in our patient with a normal set of muscle enzyme markers such as CPK. Given the negative workup for the above differentials, and with a liver biopsy consistent with DILI, we concluded that the diagnosis was HCQ-induced hepatitis with subsequent recovery after HCQ discontinuation. However, it is important to note the limitations of a case report, which include limited generalizability, lack of a control group, and data limitations.

HCQ-induced hepatotoxicity is a very rare adverse side effect that requires further investigative studies to better understand the specific pathophysiology underlying HCQ’s damaging effects on the liver. It may be advisable to follow up with patients within the first few weeks of initiating HCQ treatment in order to assess for potential signs of liver toxicity. Timely detection of HCQ-induced liver injury and early termination of HCQ will be crucial in preventing patients from progressing to fulminant hepatic failure.
